# Marked hemopneumopericardium in a patient with rectal cancer with distant metastasis: a case report

**DOI:** 10.1186/s13256-023-03935-9

**Published:** 2023-06-02

**Authors:** Sager Almulhim, Ghada Almakhaita, Emad M. AL-Osail

**Affiliations:** 1 Imam Abdulrahman Bin Faisal Hospital-National Guard, Dammam, Saudi Arabia; 2 Department of General Surgery, College of Medicine, Imam Abdulrahman Bin Faisal University, Dammam, Saudi Arabia

**Keywords:** Pneumopericardium, Hemopneumopericardium, Pericardiocentesis, Tamponade, Cancer related

## Abstract

**Introduction:**

Hemopneumopericardium defines a condition of combined pathology of weakened, dense blood content (hemopericardium) and air (pneumopericardium) in the pericardial cavity with an air fluid level. It is a rare disease, with only one such case reported in the literature. In this case report, we assessed a patient rectal cancer in addition to hemopneumopericardium, dyspnea, and chest pain.

**Clinical case report:**

A 47-year-old Arab woman previously diagnosed with rectal cancer metastasized to bones, lymph nodes, and lungs post-Hartmann procedure reported to the emergency department complaining of worsening dyspnea for 2 weeks, more significantly in the supine position. A productive cough with yellowish sputum characterized this; however, there was no pertinent family or psychological history. Examination of the respiratory system revealed dullness on the left side associated with decreased breath sound. The chest radiograph also revealed marked hydro-pneumopericardium. Spiral computed tomography angiography of pulmonary arteries demonstrated pericardial effusion with the air fluid level at pericardial space, implying hydro-pneumopericardium.

**Clinical conclusion:**

A successful pericardiocentesis was performed, in which 180 cc of blood-filled pericardial fluid was drained, suggesting the presence of hemopneumopericardium. Hemopneumopericardium has multiple etiologies, yet critical intervention is restricted in patients with cardiac tamponade. Hence, pericardiocentesis could be a definitive treatment.

## Background

Hemopneumopericardium is defined as the collection of weak, hyperdense blood content (hemopericardium) along with air (pneumopericardium) in the pericardial cavity with an air fluid level [1]. The pericardial sac commonly contains less than 50 mL of thin, clear, and straw-colored fluid. However, under various pathological conditions, the parietal pericardium may be distended by serous fluid (pericardial effusion), blood (hemopericardium), or pus (purulent pericarditis).

Pneumopericardium, a rare entity, is the presence of air in the pericardial space [2]. It can occur due to several factors such as trauma, iatrogenic, malignancy, fistulization between the pericardium and other organs, barotraumas, pericardial infections, foreign body aspiration, cocaine inhalation, aspergillosis, and diaphragmatic hernia [3, 4]. Hemopericardium is a condition of the presence of blood in the pericardial cavity. It occurs due to acute blunt or penetrating trauma from myocardial contusion, direct pericardial damage, or proximal aortic injury. It can also be found due to any chest trauma, retrograde bleeding into the pericardial sac after type I and II aortic dissection, free wall rupture after myocardial infarction, and any invasive cardiac procedure complication [5].

We present a case of hemopneumopericardium in a patient who was earlier diagnosed with rectal cancer with lung metastasis and complained of dyspnea, mostly in the supine position. Finding combined pathologies of hemopericardium and pneumocardium in a patient with cancer is rare.

## Case presentation

### Patient information

A 47-year-old Arabic woman, previously diagnosed with rectal cancer metastasized to bones, lymph nodes, and lungs (stage T4N2M1) and post-Hartmann procedure, reported to the emergency department complaining of worsening dyspnea for 2 weeks, significantly more in the supine position. There was no reported family or psychological history or any history of other medical illness, but the patient had a productive cough with yellow sputum.

### Clinical findings

**Fig. 1:**
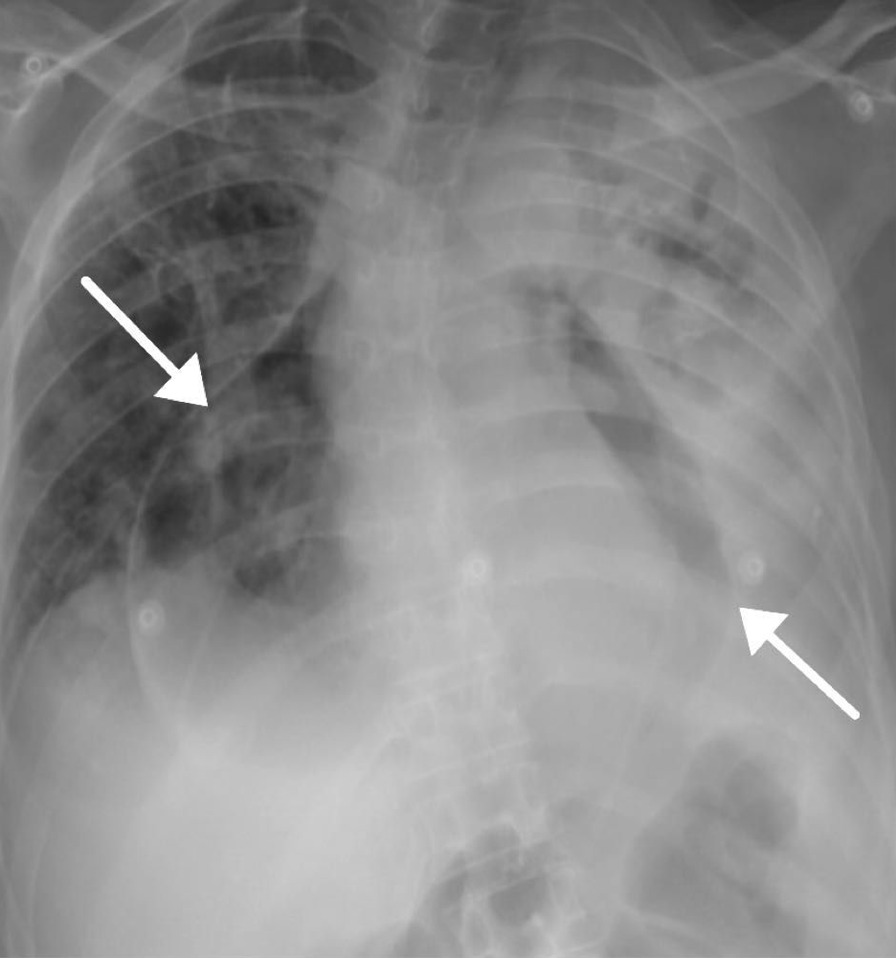
Chest radiographs revealing severe hydro-pneumopericardium, total opacification of the left lung, and air space consolidation. The arrows refer to hydro-pneumopericardium, total opacification of the left lung, and air space consolidation

**Fig. 2:**
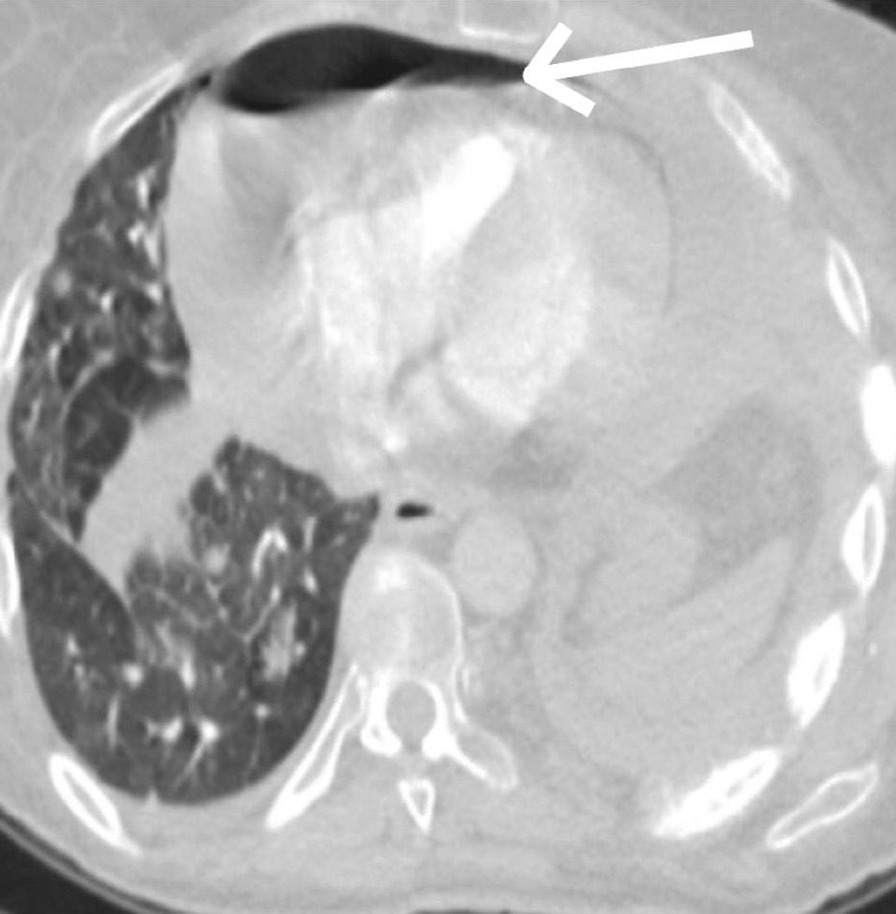
A spiral CT pulmonary angiogram showing heterogeneous soft tissue mass involving left upper and lower lung, with the mass invading the pleural surface and pericardium. The arrow refer to heterogeneous soft tissue mass involving left upper and lower lung

On clinical examination, the patient had tachypnea with a respiratory rate reaching 30, a pulse rate of 130 beats per minute, and a temperature of 37 ℃. Oxygen saturation was 95% in the O_2_ mask, she was in 2 L of oxygen, and blood pressure was within normal limits. The patient’s respiratory system examination revealed dullness associated with reduced breath sound on the left side. The rest of the physical tests were unremarkable. The laboratory test results indicated leukocytosis with a white blood cell count of 13,000/cm^2^, C-reactive protein (CRP) levels were at 116 mg/L, troponin levels at 32 ng/L, and creatinine levels at 60 μmol/L. The patient was previously diagnosed with pericardial effusion. The chest radiographs revealed severe hydro-pneumopericardium, total opacification of the left lung, and air space consolidation (Fig. 1). A spiral computed tomography (CT) pulmonary angiogram showed that there was heterogeneous soft tissue mass involving left upper and lower lung, with the mass invading the pleural surface and pericardium (Fig. 2).

### Therapeutic intervention

Emergency pericardiocentesis was planned to drain excess fluid.

**Fig. 3:**
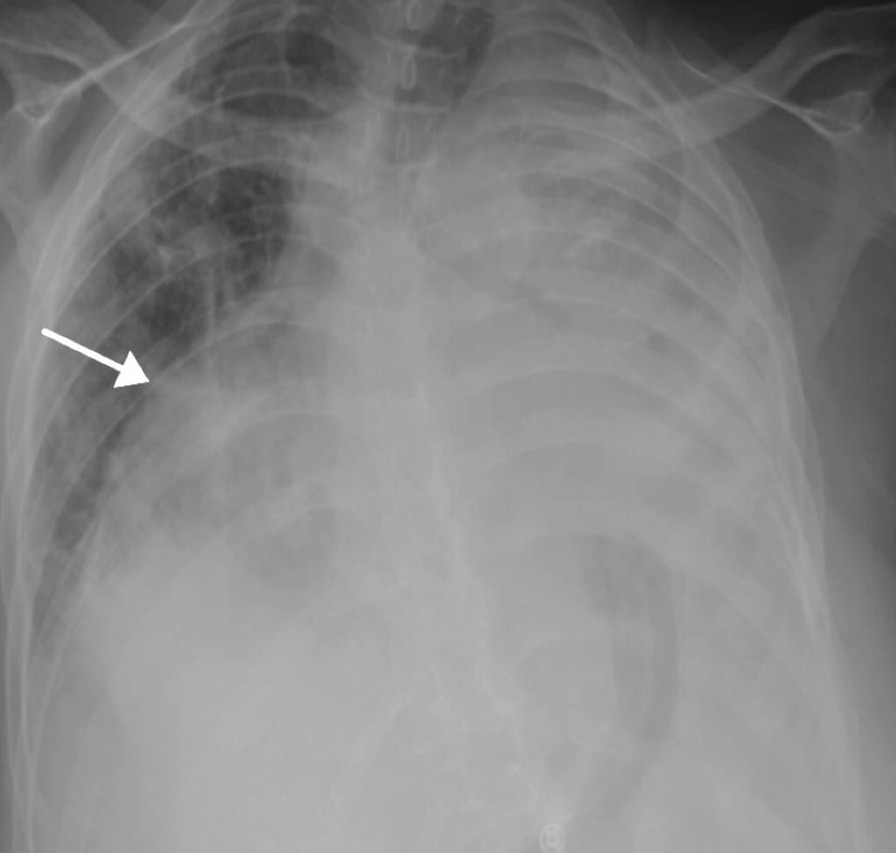
Post-intervention showing interval resolution of marked hemopneumopericardium. The arrow refer to resolution of marked hemopneumopericardium post intervention

Over 180 cc of bloody fluid was drained from pericardial sac. Pericardial fluid analysis revealed red blood cell (RBC) count of 166,400 (normal range less than 500) cells/µL, white blood cell (WBC) count of 196,800 cells/µL, neutrophils 91%, macrophages 7%, lymphocyte 2%, lactate dehydrogenase (LDH) 4054 unit/L, total protein (TP) 46 g/L, glucose 0.21 mmol/L, and high RBC count; hematocrit level 40% suggested the presence of hemopneumopericardium with no immediate complications. Cytological analysis was negative for malignancy. The pericardial tube was removed after 1 day. Post-intervention, chest radiograph revealed interval resolution of marked hemopneumopericardium. The rest of the study remains unchanged (Fig. 3). The patient was then evaluated by a cardiologist and discharged from the hospital.

### Follow-up

She was admitted for poor feeding after 1 week and discharged after 5 days. During hospitalization, a multidisciplinary meeting was conducted by the oncologists, radiologists, cardiothoracic surgeons, and pulmonologists. She was put on palliative care after expert decision. However, 3 weeks later, the patient’s primary malignancy progressed, and the patient died.

Table 1 presents a complete summary of all cases.

**Table 1:** Case summary

Age, years	47 years old
Gender	Female
Presenting complaint	Worsening dyspnea for 2 weeks associated with productive cough with yellow sputum
Physical examination	Tachypnea, respiratory system examination revealed dullness associated with reduced breath sound on the left side
Labs (abnormal results)	Leukocytosis with a white blood cell count of 13,000/cm^2^, C-reactive protein (CRP) levels of 116 mg/L
Chest X-ray	Severe hydro-pneumopericardium, total opacification of the left lung, and air space consolidation
CT scan	Heterogeneous soft tissue mass involving left upper and lower lung, with the mass invading the pleural surface and pericardium
Diagnosis	Hemopneumopericardium
Therapeutic intervention:	Emergency pericardiocentesis
Follow up after 1 week	She was admitted for poor feeding after 1 week and discharged after 5 days
Follow-up after 3 weeks	Three weeks later, the patient’s primary malignancy progressed, and the patient died

*CT* Computed tomography

## Discussion

Pneumopericardium is a rare condition that occurs owing to several reasons. Patients with pneumopericardium are generally asymptomatic; however, it can be accompanied by the back, shoulder, and epigastric pain; dyspnea; or palpitations. These symptoms depend on the progression of pneumopericardium, and the disease itself, leading to tamponade, respiratory failure, and death [6, 7].

Pneumopericardium has specific signs and includes characteristics such as auscultatory, electrocardiographic (ECG), and radiographic findings. Signs such as muffled heart sounds, shifting precordial tympany, and Hamman’s sign (crunching sound synchronous with the heartbeat due to the heart beating against air-filled tissues) can be detected during physical examination [8]. Pulsus paradoxus is commonly detected if the case is complicated by cardiac tamponade [9]. Bricheteau was normally credited with the initial clinical description of hydro-pneumopericardium in 1844. He noted the unique auscultatory findings, air and fluid coming in the pericardium, and termed it as bruit de moulin. Bricheteau also observed that each movement of the heart resulted in a sound owing to a fluctuation of fluid that was similar to “the noise made by the floats of a mill wheel as they strike the water” [10]. ST-segment elevation or depression, T-wave inversion, and low voltage are possible ECG findings [9].

Hemopericardium commonly occurs due to acute blunt or penetrating trauma from myocardial contusion, direct pericardial damage, or proximal aortic injury. This hemorrhage into the pericardial sac can rapidly cause tamponade and circulatory collapse. Hemopericardium can occur owing to any form of chest trauma, retrograde bleeding into the pericardial sac after type I and II aortic dissection, free wall rupture after myocardial infarction, and any invasive cardiac procedure complication [5]. However, when there is no trauma history, other causes such as type A aortic dissection, coronary artery aneurysm rupture, or post-infarct cardiac rupture must be considered. However, a dense pericardial effusion is not a *sine qua non* of hemopericardium or incipient tamponade. The radiographic and CT findings must be interpreted along with the history and physical examination findings. In case of features causing concern for hemodynamic compromise, echocardiography should be performed.

Pneumopericardium along with purulent fluid is exceptionally deadly. The treatment of pneumopericardium depends on the causes and stability at the hemodynamic level. In the last 12 years, approximately 11 cases of cancer-related pneumopericardium were reviewed by Samina Hirani *et al*. in 2020. Ultrasound or CT-guided pericardiocentesis is the standard gold treatment for these cases [11]. Surgical pericardiotomy and drainage are indicated in cases of recurrent pericardial effusion with a high volume of effusion and morbidity risk [12].

Hemopericardium and pneumopericardium are important markers of penetrating injury to the heart and pericardium [1]. However, a rare and unique condition of this combined pathology of hemopneumopericardium in a patient with cancer has been a case of the first of its kind. This case highlights the importance of accurately diagnosing the patient’s heart condition via a chest radiograph and CT scan, as this led to a successful pericardiocentesis to remove the blood and air from the pericardial cavity of a patient with cancer.

## Conclusion

This unique case report concluded with a condition of hemopneumopericardium in a patient with cancer, which is rare and has numerous etiologies. The diagnosis was aided by combining a CT scan and chest radiograph. However, urgent intervention is restricted in patients with cardiac tamponade. In such cases, pericardiocentesis may work as a definitive treatment.

## Data Availability

Not applicable.

## Abbreviation

CT: Computed tomography
CRP: C-reactive protein
ECG: Electrocardiographic
